# Ultrastructural Alterations of Myelinated Fibers and Oligodendrocytes in the Prefrontal Cortex in Schizophrenia: A Postmortem Morphometric Study

**DOI:** 10.1155/2011/325789

**Published:** 2011-06-28

**Authors:** Natalya A. Uranova, Olga V. Vikhreva, Valentina I. Rachmanova, Diana D. Orlovskaya

**Affiliations:** Laboratory of Clinical Neuropathology, Mental Health Research Center, Zagorodnoe shosse 2, Moscow 117152, Russia

## Abstract

Schizophrenia is believed to result from altered neuronal connectivity and impaired myelination. However, there are few direct evidence for myelin abnormalities in schizophrenia. We performed electron microscopic study of myelinated fibers and oligodendrocytes and morphometric study of myelinated fibers in the prefrontal cortex in gray and white matters in schizophrenia and normal controls. Six types of abnormal fibers and ultrastructural alterations of oligodendrocytes were found in schizophrenia. No significant group differences in area density of myelinated fibers were found. Frequency of pathological fibers was increased significantly in gray matter in young and elderly schizophrenia patients and in patients with predominantly positive symptoms. In contrast, in white matter, frequency of altered fibers was increased significantly in elderly patients, in patients with predominantly negative symptoms, and correlated with illness duration. Progressive alterations of myelinated fibers in white matter might be followed by alterations of myelinated fibers in gray matter in schizophrenia.

## 1. Introduction

Growing evidence coming from neuroimaging and postmortem studies support the notion that altered neuronal connectivity in schizophrenia is associated with disturbed myelination in different fiber tracts [1–9]. Abnormalities of oligodendrocytes in gray and white matters in schizophrenia are among the most consistent findings. Myelin and oligodendrocyte abnormalities have been reported in schizophrenia in neuroimaging, neurocytochemical, microarray, and morphometric studies [1]. 

*In vivo* neuroimaging studies demonstrated reduced white matter volume in the prefrontal cortex [10]. Buchsbaum et al., [11] in PET study, found an increased relative metabolic rates in white matter in schizophrenia. Metabolic abnormalities in fronto-striatal-thalamic white matter tracts have been reported in schizophrenia [12]. MR diffusion tensor imaging (DTI) can measure and visualize organization of white matter fiber tracts *in vivo*. DTI studies of white matter in schizophrenia demonstrated a significant reduction in fractional anisotropy (FA) in frontal lobe and the relationships between white matter abnormalities and symptoms of the disorder [13–16]. It is not known whether the abnormalities observed to date reflect a decrease in number of axons, decreased axonal diameter, thinner myelin sheaths, or less coherent fibers. 

Morphometric studies revealed prominent deficit of oligodendrocytes in different brain regions, including gray and white matter of the prefrontal cortex in schizophrenia [17–22]. The first stereological study estimated the length of myelinated nerve fibers in the whole brain, as well as in the prefrontal cortex of subjects with schizophrenia did not find significant group differences [23]. Postmortem electron microscopy revealed ultrastructural alterations of myelinated nerve fibers, damage and degeneration of oligodendrocytes in layer VI of the prefrontal cortex in schizophrenia [24]. Dysfunction and reduced proliferation of these cells were proposed to explain these findings. Oligodendrocytes were found to be the most severely affected cell type in schizophrenia and bipolar disorder as compared to normal controls [24], and deficit of oligodendrocytes was found in layers V and VI of the prefrontal BA10 and in underlying white matter [21, 22]. However, it remains unknown whether alterations of myelinated fibers in white matter differ from those in gray matter, whether myelin abnormalities are associated with aging, schizophrenia, and medication effects. 

Previously, we reported alterations of myelinated fibers in layer V of the prefrontal cortex in schizophrenia using small sample size [25]. Here, we present (1) the results of qualitative and morphometric studies of myelinated fibers in layer V of the prefrontal cortex, using rather big sample size, and in adjacent white matter in schizophrenia as compared to normal controls and (2) the results of qualitative study of oligodendrocytes in gray and white matters in schizophrenia as compared to normal controls. Since deficit of oligodendrocytes was found both in gray matter of the prefrontal BA10 and in underlying white matter in the subgroup of schizophrenia subjects with predominantly negative symptoms [21, 22], we expected more severe alterations of myelinated fibers in this subgroup as compared to the subgroup with predominantly positive symptoms.

The aim of the study was to detect ultrastructural abnormalities of myelinated fibers and oligodendrocytes in gray matter of the prefrontal cortex (layer V, BA10) and in underlying white matter in schizophrenia. Our study addressed the following questions: (1) What kind of ultrastructural abnormalities of myelinated fibers and oligodendrocytes differ schizophrenia subjects from controls, particularly, whether an axonal degeneration and loss of myelin occur in schizophrenia? (2) Do the ultrastructural alterations of myelinated fibers in gray matter differ from those in white matter? (3) Are the changes of myelinated fibers associated with age, gender, age at onset, and duration of illness? (4) Is there a link between abnormalities of myelinated fibers and symptoms of schizophrenia and neuroleptic exposure?

## 2. Materials and Methods

### 2.1. Materials

The study was performed using the Mental Health Research Center (MHRC) brain collection. Prefrontal cortex (BA10, layer V) was studied in 40 cases of schizophrenia and 40 normal matched controls with a short postmortem delay. White matter underlying prefrontal BA10 was studied in 12 cases of schizophrenia and 12 normal controls available. Clinical records were obtained. ICD-10 and DSM-IV-R diagnoses were made by psychiatrists from MHRC. The Scale for the Assessment of Negative Symptoms (SANS) and the Scale for the Assessment of Positive Symptoms (SAPS) were used by psychiatrists to rate negative and positive symptoms during the last hospitalization in schizophrenia subjects. Summary scores of negative and positive symptoms were determined on the basis of the ratio of the percentage of negative and positive scores in summary scores. Basic demographic and clinical data are given in Table 1. Cases were coded for morphometric blind study.

### 2.2. Methods

#### 2.2.1. Electron Microscopy

For electron microscopy, small tissue pieces from gray matter of BA10 (left hemisphere) and from underlying white matter were obtained perpendicular to cortical surface. The tissue pieces were fixed by immersion with mixture of 2,5% glutaraldehyde and 4% paraformaldehyde in 0,1 M phosphate buffer for 1 week, then postfixed in 1% osmium tetroxide for 1 hour, stained with uranyl acetate for 1 hour, dehydrated in ethanol series and embedded in Araldit epoxy resin. Sections were cut using Reichert ultramicrotome, and semithin 1 *μ*m sections stained with toluidine blue were used for orientation in cortical layers. Small pyramids were trimmed on layer V (layer of big pyramidal cells) and on adjacent white matter. Ultrathin sections were cut, put on formvar-coated, slot-type grids, counterstained with lead citrate and viewed with the electron microscope Philips EM 420.

#### 2.2.2. Light Microscopy

Besides, for all the cases, histological standard methods were used: Nissl staining, hematoxilin-eosin staining, Van Gieson staining, Bielshowsky, and Congo red. No histological evidence of neurodegenerative, inflammatory, and ischemic diseases were found.

#### 2.2.3. Morphometry

Morphometric study was performed to estimate the number of myelinated fibers per unit tissue area and the frequency of normally myelinated and pathological myelinated fibers in control and schizophrenia groups. 3 tissue blocks from layer V and 3 tissue blocks from white matter per each case were randomly selected. Systematic random sampling was used to estimate the number of myelinated axons present. The starting point for making photos was determined as the leftward top point of the section. Series of photographs were made at the distance 40 microns until the rightward edge of the section was reached. The camera was then moved 40 microns down, and photos of the myelinated fibers were taken in the same manner until the leftward edge of the section was reached. This process was repeated until 45 sampling fields in layer V and 48 sampling fields in white matter from each subject were systematically sampled and photographed under 4,000x magnification. Mean sample area per case was 14,850 *μ*m^2^ in gray matter and 15,800 *μ*m^2^ in white matter. Mean number of myelinated fibers per case counted in gray matter was 1,495 in control group and 1,438 in schizophrenia group; in white matter, it was 4,560 in control group and 4,056 in schizophrenia group. EM images of myelinated fibers were superimposed on the enlarger “Minolta” to get the final magnification 26,000x. 

To quantify myelinated fibers, transversely sectioned nerve fibers were examined. Area density of myelinated fibers and the frequency of normal and pathological myelinated fibers were estimated. Area density of myelinated fibers was calculated as the number of fibers per unit tissue area (1000 *μ*m^2^). The frequency of normal and pathological myelinated fibers present was calculated as the number of normal or pathological fibers in unit tissue area per number of total myelinated fibers in unit tissue area expressed in percentages.

#### 2.2.4. Statistical Analyses

Statistical analysis was performed using STATISTICA 6 software package for Windows (StatSoft Inc, Tulsa, OK, USA). Area density of myelinated fibers, frequency of normal and all pathological myelinated fibers, and frequency of different types of pathological fibers were analyzed.

Correlation analysis was performed for control and schizophrenia group to examine the influence of postmortem delay, age, duration of disease, medication (expressed as chlorpromazine equivalents according to Davis [26]), and correlations between the parameters measured. Since the normal distribution of the data by Kolmogorov-Smirnov tests was obtained, the Pearson correlation coefficient was used. Age effects were also analyzed using one-way analysis of variance (ANOVA) comparison of young (≤45 y.o.) and elderly (>45 y.o.) control and schizophrenia patients taking Bonferroni correction. 

Differences between control group and schizophrenia group, between control group and schizophrenia clinical subgroups of subjects with predominantly positive or predominantly negative symptoms were examined. Also these clinical subgroups were compared. 

Group comparisons were made using one-way ANOVA. Differences between the subgroups with predominantly positive or predominantly negative symptoms were examined using one-way ANOVA, followed by post hoc test, taking the Bonferroni correction. Two-way ANOVA followed by post hoc test with the Bonferroni correction was used to detect gender effects.

## 3. Results

### 3.1. Gray Matter

#### 3.1.1. Qualitative Study

**Figure 1:**
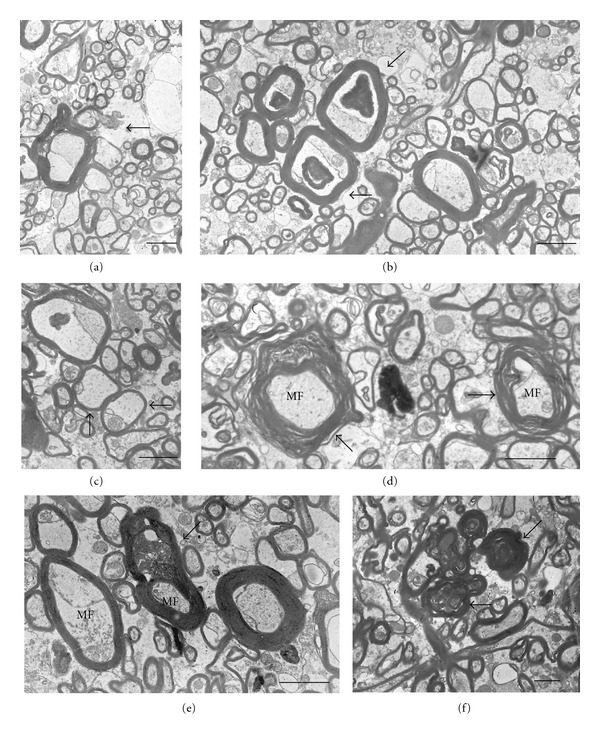
Six types of pathological myelinated fibers. Descriptions in the text. Scale bars  =  2 *μ*m.

Qualitative study demonstrated that the ultrastructure of myelinated nerve fibers was well preserved in both schizophrenia and control subjects. The study of myelinated fibers revealed six types of pathological fibers demonstrating ultrastructural alterations of myelinated fibers in schizophrenia subjects and in controls (Figure 1). Myelinated fibers of type 1 (P1) were characterized by focal lysis of myelin sheath lamellae, sometimes with the formation of concentric lamellar bodies. This type of pathology often appeared in close apposition to astrocytic processes or inside them (Figure 1(a)). Fibers of type 2 (P2) exhibited the presence of myelin-like figures of unknown origin in swollen periaxonal oligodendroglial processes though myelin sheaths looked well preserved. (Figure 1(b)). Type 3 (P3) fibers demonstrated swelling of periaxonal oligodendroglial processes, atrophy of inner axon, and preserved myelin sheath. These types of alterations of myelinated fibers were seen mostly in myelinated fibers of small caliber, containing few myelin sheath lamellae (Figure 1(c)).

Besides, three types of degenerating myelinated fibers were rarely seen in gray matter in schizophrenia and controls. They demonstrated dark degeneration of myelinated fibers (type 4, P4) (Figure 1(f)), degeneration of myelin sheaths: splitting and decompaction of myelin sheath lamellae (type 5, P5) (Figure 1(d)), and inclusions of dense cytoplasm with vacuoles resembling the cytoplasm of microglial cell between myelin sheath lamellae (type 6, P6) (Figure 1(e)). Importantly to note is that all these types of pathological alterations of myelinated fibers demonstrated deformations of fibers (Figure 1). P4–6 fibers were seen mostly in white matter, in gray matter they were observed very rarely in schizophrenia and in controls, so they were counted only in white matter.

**Figure 2:**
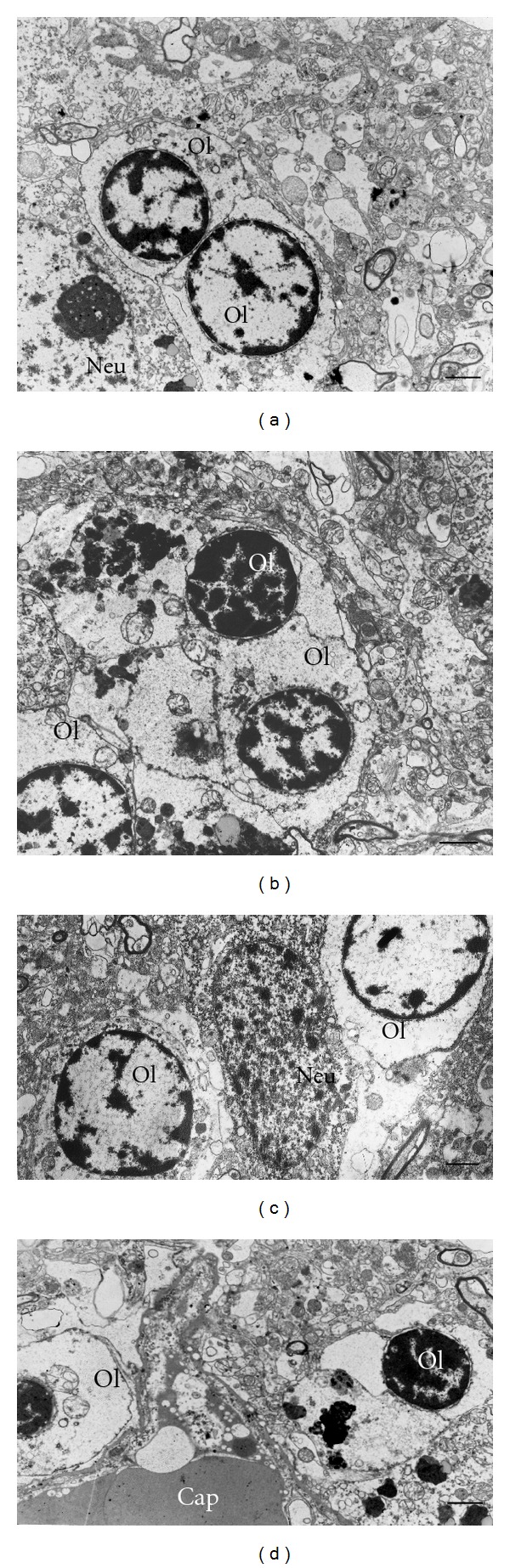
Electron micrographs of oligodendrocytes in gray matter from control subject (a) and from subjects with schizophrenia (b–d). Dystrophic alterations of oligodendrocytes, Ol, (b, c) and destructive changes of oligodendrocytes (d). Scale bars  = 2 *μ*m.

The changes of myelinated fibers were accompanied by prominent alterations of the ultrastructure of oligodendrocytes in schizophrenia as compared to controls (Figure 2). Electron lucent chromatin and small rim of cytoplasm were characteristic features of oligodendrocytes in control brains (Figure 2(a)). In schizophrenia subjects, oligodendrocytes demonstrated dystrophic and destructive alterations. The most prominent changes were chromatin condensation (Figures 2(b) and 2(d)) and swelling of cytoplasm (Figures 2(b)–2(d)). Destructive alterations of oligodendrocytes were rarely seen in control brains.

#### 3.1.2. Morphometry

**(1) Effects of Disease and Age**

Morphometric study showed that the area density of myelinated fibers did not differ significantly: (mean ± SEM) 101 ± 6,0 per 1000 *μ*m^2^ in control group and 105 ± 6.4 per 1000 *μ*m^2^ in schizophrenia group.

**Figure 3:**
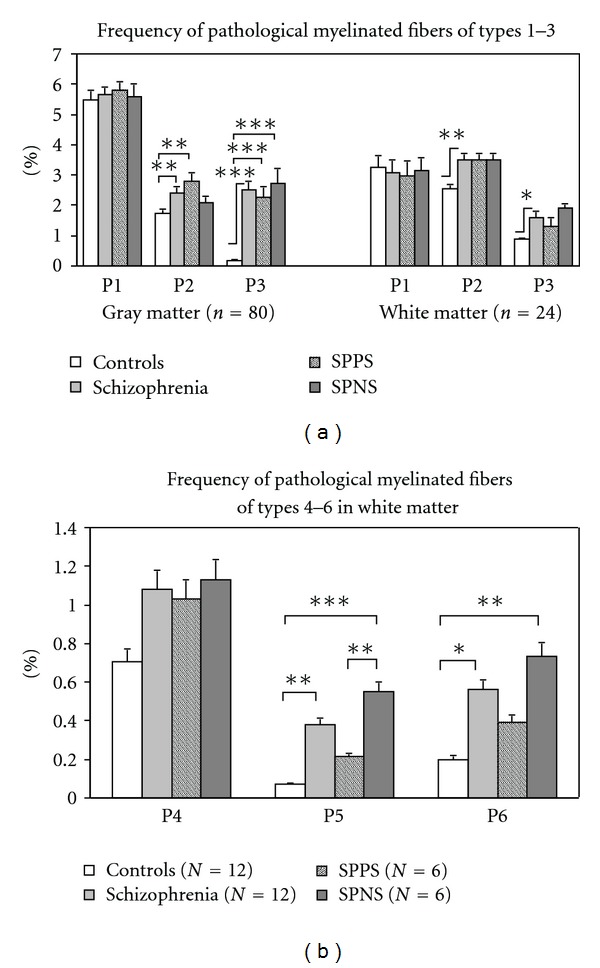
Frequency of pathological myelinated fibers present in gray matter and in white matter (mean ± SEM). SPPS: schizophrenia subjects with predominantly positive symptoms. SPNS: schizophrenia subjects with predominantly negative symptoms. *P < .05, **P < .01, ***P < .001.

Frequency of all pathological myelinated fibers was significantly increased in schizophrenia group as compared to the control group (*F* = 12.9, *df* = 1.78, *P* = .0006). Frequency of P1 fibers did not differ significantly from controls. A significant increase in the frequency of both P2 fibers (*F* = 8.6, *df* = 1.78, *P* = .004) and P3 fibers were found in schizophrenia as compared to controls (*F* = 62.6, *df* = 1.78, *P* < .0001). (Figure 3(a)).

Comparison of the subgroups with predominantly positive symptoms, predominantly negative symptoms, and the control group demonstrated a significant differences in the frequency of all pathological myelinated fibers (*F* = 6.25, *df* = 2.75, *P* = .003) as well as in the frequencies of P2 fibers (*F* = 6.49, *df* = 2.75, *P* = .0025) and P3 fibers (*F* = 30.3, *df* = 2.75, *P* < .0001). Post hoc showed that the frequency of all pathological myelinated fibers and of P2 fibers significantly increased in the subgroup of patients with predominantly positive symptoms (*P* = .002). The frequency of P3 fibers increased both in the subgroup with predominantly positive symptoms (*P* = .0001) and with predominantly negative symptoms as compared to controls (*P* = .0001) (Figure 3(a)).

The frequency of P2 fibers correlated negatively and significantly with the frequency of P3 fibers in schizophrenia group (*r* = −0.36, *P* < .05) in contrast to the control group where no significant correlations were found (*r* = −0.06, *P* = .7).

**Figure 4:**
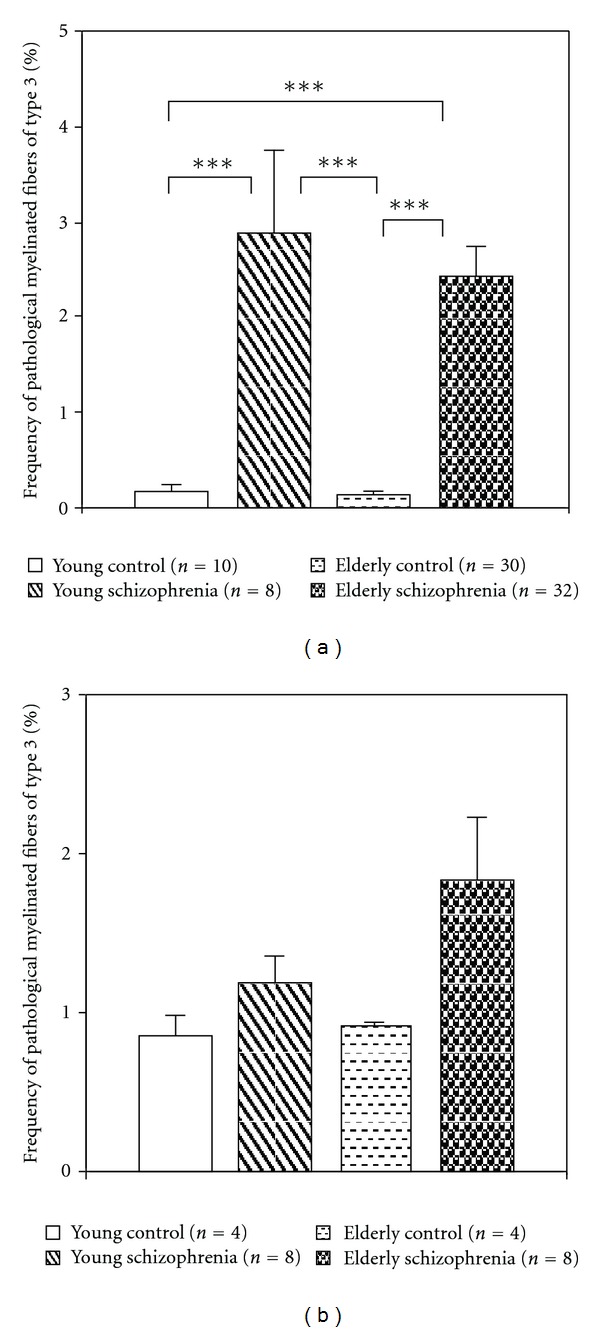
Histograms demonstrating the results of comparisons of the frequency of pathological myelinated fibers of type 3 in young and elderly patient subgroups with young and elderly controls. (a) Gray matter, (b) White matter (mean ± SEM).

No correlations with age of the estimated parameters were found in schizophrenia and control groups. However, comparison of young (≤45 y.o.) and elderly (>45 y.o.) subgroups of patients with young and elderly control subjects demonstrated that both young and elderly schizophrenia patients differed from two control subgroups in the frequency of P3 fibers (Figure 4(a)). There were no significant differences between the age subgroups in other types of fibers.

We did not find the effects of gender in either control or schizophrenia group. There were no correlations between the parameters of myelinated fibers and age at onset or duration of schizophrenia.

**(2) Effects of Other Confounding Variables**

We did not find the effects of postmortem delay and neuroleptic exposure on the frequency of myelinated fibers.

### 3.2. White Matter

#### 3.2.1. Qualitative Study

Three types of myelinated fibers described in detail in gray matter were also seen in the white matter (Figure 1). Besides, degenerating myelinated fibers were observed in white matter, and they demonstrated dark degeneration of myelinated fibers (type 4, P4) (Figure 1(f)) and degeneration of myelin sheaths of two types: splitting of myelin sheath lamellae (type 5, P5) (Figure 1(d)) and inclusions of dense cytoplasm with vacuoles resembling the cytoplasm of microglial cell in between myelin sheath lamellae (type 6, P6) (Figure 1(e)). The ultrastructural pathological findings were present mostly in large- and medium-sized myelinated fibers.

**Figure 5:**
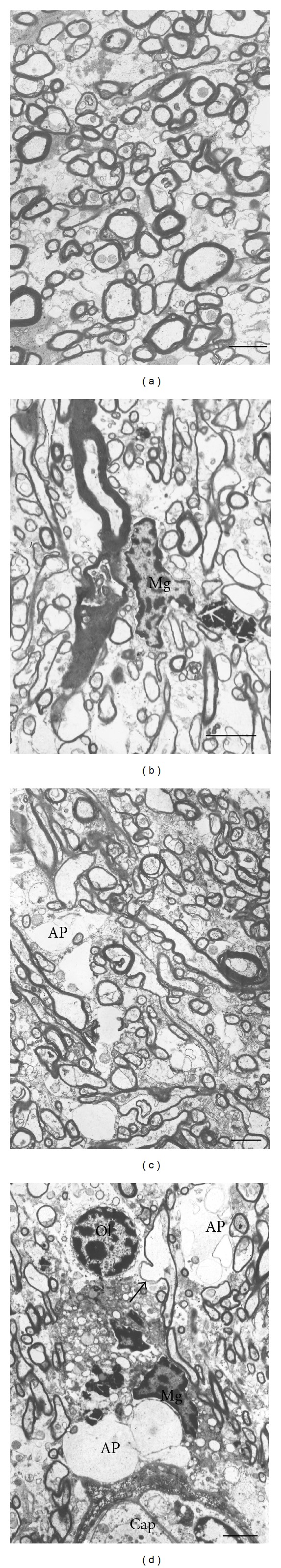
Electron micrographs of white matter from control brain (a) and from the brains of subjects with schizophrenia (b–d). Both thinly myelinated (b, c) and axons with focal lysis of myelin sheath (d) as well as some axons with abnormally thick myelin sheaths (Figure 1(b)) were observed in schizophrenia in contrast to controls. Ap: astrocytic process, Mg: microglial cell. Ol: oligodendrocyte. Scale bars = 2 *μ*m.

 Myelinated fibers in white matter varied in size in control (Figure 5(a)) and schizophrenia brains (Figures 5(b)–5(d)). However, in schizophrenia brains both thinly myelinated (Figure 5(b)), axons with focal lysis of myelin sheath (Figure 5(d)) as well as some axons with abnormally thick myelin sheaths (Figure 5(b)), were often observed in contrast to controls. The second important finding was that myelinated fibers were often located in close apposition to numerous swollen astrocytic processes in schizophrenia subjects (Figures 5(c) and 5(d)) in contrast to controls where such swollen processes were not observed (Figure 5(a)).

**Figure 6:**
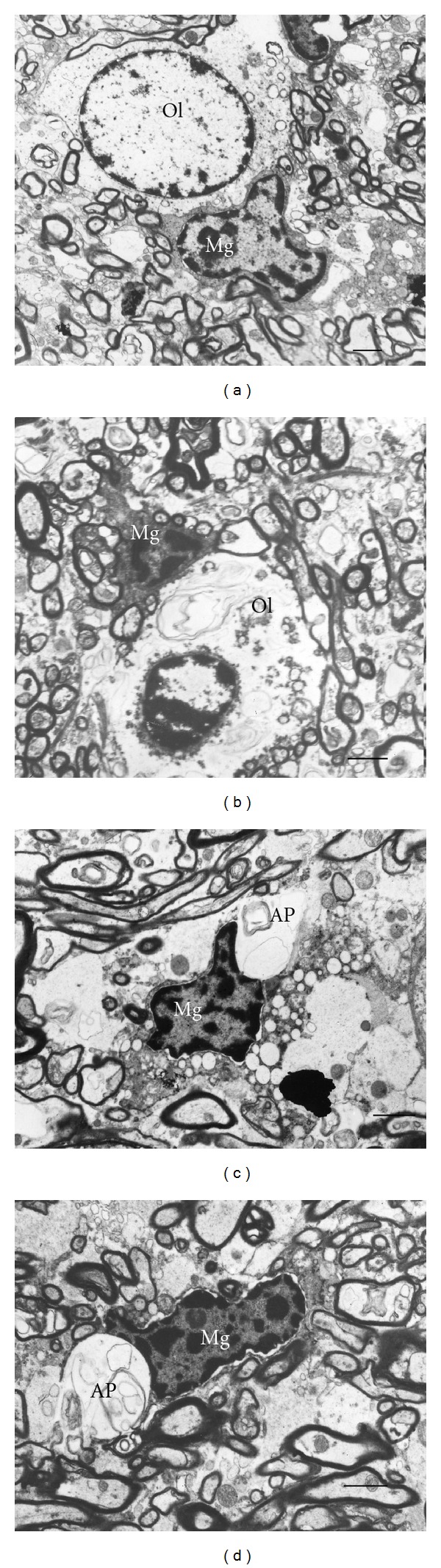
Electron micrographs of oligodendrocytes and microglia in white matter from control subjects (a) and from subjects with schizophrenia (b–d). Dystrophic changes of oligodendrocyte, Ol, (b). Activated microglia in close apposition to swollen astrocytic process (Ap) containing myelin debris in schizophrenia brain (c) Microglial cell participates in phagocytosis of myelin membranous debris in schizophrenia (d). Scale bars = 2 *μ*m.

The alterations of myelinated nerve fibers in white matter were accompanied by prominent dystrophic changes of oligodendrocytes in schizophrenia brains but not in controls. Oligodendrocytes in subjects with schizophrenia looked swollen and vacuolated as compared to controls (Figures 6(a) and 6(b)). No destructive changes of oligodendrocytes were found in white matter in either schizophrenia or control brains. Microglial cells were often located in close apposition to oligodendrocytes in control subject (Figure 6(a)) and in schizophrenia subject (Figure 6(b)) and apposed to myelinated fibers in schizophrenia subject (Figure 6(b)). Activated microglial cells containing invaginated nucleus and vacuolated cytoplasm were observed in close apposition to swollen astrocytic processes containing membranous debris (Figure 6(c)). Microglial cell participates in phagocytosis of myelin membranous debris in schizophrenia (Figure 6(d)). This type of activation of microglial cells was not seen in control subjects.

#### 3.2.2. Morphometry

**(1) Effects of Disease and Age**

Morphometric study showed that the area density of myelinated fibers was nonsignificantly decreased (−7%) in schizophrenia: 323 ± 29.07 per 1000 *μ*m^2^ as compared to normal controls: 345 ± 31,1 per 1000 *μ*m^2^.

Frequency of all pathological myelinated fibers increased in schizophrenia as compared to controls (*F* = 5.33, *df* = 1.22, *P* = .03). Among them, frequency of P1 fibers was nonsignificantly increased, frequency of P2 and P3 fibers increased significantly (Figure 3(a)), frequency of P4 fibers tended to increase (*P* = .08), and frequency of P5 and P6 fibers increased significantly (Figure 3(b)). The frequency of P2 fibers was not correlated with the frequency of P3 fibers in either schizophrenia or control group in contrast to gray matter where these parameters significantly correlated.

Comparison of the subgroups with predominantly positive symptoms, predominantly negative symptoms, and control group showed a significant differences in the frequency of P3 (*F* = 3,6, *df* = 2,21, *P* < .05), P5 (*F* = 11.05, *df* = 2.21, *P* < .001) and P6 (*F* = 5.45, *df* = 2.21, *P* = .01) fibers. Post hoc demonstrated a significant increase in the frequency of P5 (*P* = .0004) and P6 myelinated fibers in the subgroup with predominantly negative symptoms (*P* = .006) (Figure 3(b)). Also, frequency of P5 fibers was significantly higher in the subgroup with predominantly negative symptoms as compared to the subgroup with predominantly positive symptoms (*P* = .005).

The frequency of P1 and P6 fibers correlated significantly with age in schizophrenia group (*r* = 0.74, *P* < .01, *r* = 0.62, *P* < .05, resp.) but not in the control group, where only frequency of P5 correlated significantly with age (*r* = 0.6, *P* < .05). No other correlations of myelinated fibers with age were found. Comparison of young (≤45 y.o.) and elderly (>45 y.o.) subgroups of patients with young and elderly control subjects showed no significant differences between the subgroups in P3 fibers in contrast to significant changes of the parameter in gray matter (Figure 4). No significant differences between the age subgroups in other types of fibers were found. The frequency of pathological fibers was not correlated with age at the onset of disease.

The effects of gender were not found in the groups studied.

**(2) Effects of Other Confounding Variables**

**Figure 7:**
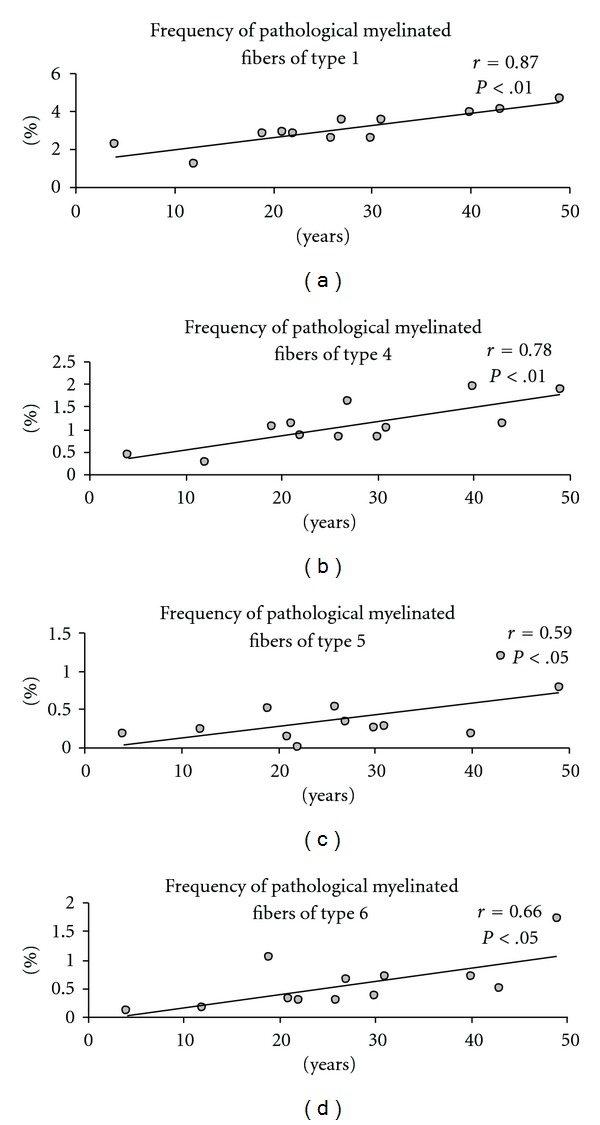
The association between frequency of pathological myelinated fibers in white matter and duration of schizophrenia.

We did not find the effects of postmortem delay and neuroleptic exposure on the parameters of myelinated fibers. However, the effect of duration of disease was found. The frequency of pathological myelinated fibers (P1, P4–6) correlated positively and significantly with illness duration (Figure 7).

## 4. Discussion

The present study provides evidence for alterations of oligodendrocytes and myelinated nerve fibers in the prefrontal cortex in schizophrenia as compared to controls. Comparative study of oligodendrocytes and myelinated nerve fibers in gray matter and in white matter showed both similarities and differences between gray matter and white matter in schizophrenia. In gray matter, dystrophic and destructive changes of oligodendrocytes were observed in schizophrenia, and in white matter, only dystrophic changes of oligodendrocytes were found in schizophrenia. Morphometric study showed damage of myelinated fibers including altered oligodendrocyte/axon interaction and myelin/axon integrity, axonal atrophy, and focal damage of myelin sheaths in gray matter in schizophrenia as compared to controls. The same changes were found in white matter in schizophrenia. Besides, frequency of degenerating myelin sheaths was significantly increased in white matter, while in gray matter they were rarely observed. In white matter, both astrocytic cell processes and microglial cells were involved in the phagocytosis of degenerating myelin sheaths in schizophrenia. Since myelin is produced by oligodendrocytes, these data suggest that the pathology of myelinated nerve fibers in schizophrenia might be due to oligodendrocyte abnormalities. The data are consistent with the results of our previous studies reported ultrastructural changes and deficit of oligodendrocytes in lower layers of the prefrontal BA10, and in adjacent white matter in schizophrenia [21, 22, 24] and with prominent deficit of oligodendrocytes in gray and white matters of the dorsolateral prefrontal cortex in schizophrenia [17, 18], though Segal et al. [27] found no significant differences in the oligodendrocyte distribution or density in the cingulum bundle in schizophrenia. However, the origin of the differences between gray and white matter abnormalities detected in schizophrenia remains uncertain. 

We did not find significant changes in area density of myelinated fibers in either gray matter or in white matter in schizophrenia. It is important to note that there were no differences in the number and density of fibers in the uncinate fasciculus [28] and in the length of myelinated nerve fibers in the whole brain, as well as in the prefrontal cortex [23] reported in schizophrenia. 

Frequency of normal fibers was decreased, and the frequency of pathological fibers was increased significantly in gray matter in young and elderly schizophrenia patients. In contrast, frequency of pathological fibers in white matter was increased significantly in elderly patients. These data suggest that alterations of myelinated fibers in white matter in schizophrenia might be followed by alterations of myelinated fibers in gray matter. 

*Do the alterations of myelinated fibers progress in the course of illness? *In white matter, significant correlations of the frequency of pathological myelinated fibers with duration of illness were found in schizophrenia subjects. The results are in agreement with the progressive frontotemporal gray matter reduction and frontoparietal white matter expansion in schizophrenia associated with poor outcome during a chronic stage of illness [29] Mori et al. [30] found in white matter a significant negative correlation between FA and duration of illness. Progressive white matter loss may be a consequence of chronic disease [31–33]. We consider that the changes found in white matter in the present study might be a consequence of chronic disease including dystrophic and degenerative processes in oligodendrocytes and myelin sheaths. Degenerating myelin sheaths were found in medium and large-sized myelinated fibers suggesting that cortico-subcortical and cortico-cortical fibers might be involved in degenerating process of myelinated fibers in schizophrenia. 

*Do myelin alterations contribute to symptoms of schizophrenia? *The present study demonstrated that in gray matter, the frequency of pathological fibers was increased significantly in the subgroup of patients with predominantly positive symptoms. On the contrary, in white matter the frequency of myelinated fibers containing degenerating myelin sheaths was increased significantly in subjects with predominantly negative symptoms. It is important to note that according to our previous data, cases with predominantly negative symptoms showed significant increase in the volume fraction of heterochromatin in layer VI, prominent deficit of pericapillary oligodendrocytes in layer V of the prefrontal BA10 and deficit of oligodendrocytes in adjacent white matter in schizophrenia [21, 22, 24]. These results also suggest that there is a link between alterations of oligodendrocytes and of myelinated fibers in schizophrenia. 

Our data are in accordance with the results of neuroimaging studies that prefrontal white matter FA correlated with negative symptoms, impulsiveness, and aggressiveness [34, 35]. Reductions in prefrontal white matter may be associated with schizophrenia-negative symptoms [36]. Wible et al. [37] reported that schizophrenia subjects with high negative symptom scores had significantly smaller bilateral white matter volumes than those with low negative symptom scores. Decrements in prefrontal white matter related to higher levels of negative symptoms [38], increased density of interstitial white matter neurons in dorsolateral prefrontal cortex in the deficit group compared with the nondeficit as well as the control [39] have been reported in schizophrenia patients. Negative symptoms may involve disruption of frontal-subcortical connections [40]. 

White matter might play a role in producing cognitive impairment in schizophrenia [6]. Cognitive deficit in schizophrenia is associated with the dysfunction of the prefrontal cortex [38, 41]. Myelinated nerve fiber degeneration plays a role in the pathogenesis of age-related cognitive decline [42]. In area 46 of monkey, the age-related alterations of myelinated fibers significantly correlate with the cognitive impairment index [42]. It is proposed that age-related correlations between frequency of myelin alterations and impairments in cognition occur because the conduction velocity along the affected nerve fibers is reduced, so that the normal timing sequences within neuronal circuits break down [42–45]. Our study detected positive correlation between age and the frequency of degenerating myelin sheaths in the white matter in control group (P5 myelinated fibers) and in schizophrenia group (P6 myelinated fibers), similar to those described in monkey during aging and correlated with cognitive impairment [46], though the effect of illness duration was more pronounced. These data suggest that degeneration of myelin sheaths in white matter found in the present study might be associated with cognitive impairment in schizophrenia.

*Disturbed oligodendrocyte-axon relationships in schizophrenia. *Our results support the hypothesis of Mitterauer [47] that decomposition of the oligodendrocyte-axonic system may be responsible for symptoms of incoherence (thought disorder, etc.) in schizophrenia. Our study detected prominent dystrophic alterations of oligodendrocytes, including their swelling in both gray matter and in white matter in schizophrenia, and destructive changes of oligodendrocytes in gray matter in schizophrenia. These data are in accordance with the most pronounced changes of P3 myelinated fibers in schizophrenia demonstrating swelling of periaxonal oligodendrocytic process and shrinkage of inner axon of myelinated fibers. Besides, the frequency of the P2 fibers in gray matter correlated negatively and significantly with the frequency of P3 fibers in schizophrenia group in contrast to control group where no significant correlations were found. These data suggest that the P2 and P3 types of pathological myelinated fibers are interrelated. They might present a common type of alterations of myelinated fibers that might lead to axonal atrophy. P3 myelinated fibers had small size with thin myelin sheath. The data suggest that this type of pathological myelinated fibers might belong mostly to associative fibers or to axons of interneurons. A significant increase in the frequency of this type of myelin pathology has been reported in the upper layers of the prefrontal and visual cortices [48], in the hippocampus [49] and in the caudate nucleus [25] in schizophrenia. The results are consistent with the ultrastructural damage of oligodendrocytes in the hippocampus [49] and in the caudate nucleus [24] in schizophrenia. The data suggest a widespread axonopathy due to injured oligodendrocyte-axon interactions in schizophrenia brains. These changes of myelinated fibers might cause changes in conduction velocity and might lead to the atrophy of presynaptic axon terminals reported in the prefrontal cortex in schizophrenia [50] and may contribute to altered connectivity in schizophrenia [51]. 

Haroutunian et al. [52] has reported that some of the genes affected in schizophrenia are associated with the regulation of axoglial contacts, axon calibre, and the integrity of functional elements involved in signal propagation. In KCC3 knockout mice, an animal model of agenesis of the corpus callosum associated with peripheral neuropathy, some fibers accumulate fluid periaxonally. The swelling pathologies are followed by axon and myelin degeneration in adult nerves, leading to reduction in nerve conduction velocity [53]. To maintain axonal integrity, mammalian myelin-forming cells require the expression of some glia-specific proteins, including CNP, PLP, and MAG, as well as intact peroxisomes, none of which is necessary for myelin assembly. Loss of oligodendroglial support causes progressive axon degeneration and possibly local inflammation, both of which are likely to contribute to a variety of neuronal diseases in the central and peripheral nervous systems [54]. Axoglial interactions underlie the clustering of ion channels and of cell adhesion molecules, regulate gene expression, and control cell survival. Rasband et al. [55] reported that Cnp1-null mice, lacking expression of the myelin protein cyclic nucleotide phosphodiesterase (CNP), have disrupted axoglial interactions in the central nervous system. However, Mitkus et al. [56] did not find altered expression of MAG, CNP, and OLIG2 in the gray or white matter in patients with schizophrenia.

Benes [57] hypothesized delayed myelination in the prefrontal cortex in patients with schizophrenia. Abnormalities of myelination in the frontal cortex in schizophrenia were detected *in vivo* with MRI, and postmortem with analysis of oligodendrocyte proteins [2, 58–62]. Decreased expression of the glial gene *Quaking (QKI)*, encoding an RNA binding essential for myelination, has been reported in schizophrenia brain. These data support the notion that dysmyelination occurs in the brain of patients with schizophrenia. 

MRS studies of patients with chronic schizophrenia as well as at first episode prior to treatment showed alterations in neuronal membrane biochemistry, including the prefrontal cortex [63]. The results of both metabolomic and proteomic studies pointed to energy metabolism and lipid biosynthesis [64], alterations of free fatty acids, phosphatidylcholines, and ceramides [65], cytoskeleton, oligodendrocyte, energy metabolism and cell-signalling proteins being impaired in schizophrenia [66]. Tkachev et al. [67] provide evidence for altered myelin biosynthesis and glutamatergic dysfunction in the prefrontal cortex in schizophrenia. Cerebral white matter, composed of myelin-containing oligodendrocytes, is highly sensitive to glutamate excitotoxicity, abnormally expressed NMDA receptor subunits in elderly patients with schizophrenia [68]. NMDA receptors have been shown to have an important role in mediating Ca^2+^–dependent injury of oligodendrocytes and the myelin sheath. Together, these data suggest that oligodendrocytes and myelin abnormalities might be among the major components of the neurobiology of schizophrenia.

## 5. Conclusions

Abnormalities of myelinated fibers in schizophrenia include altered oligodendrocyte/axon interaction and myelin/axon integrity, axonal atrophy, damage of myelin sheaths in gray matter and in white matter, and degeneration of myelinated sheaths in white matter. Alterations of myelinated fibers in schizophrenia are accompanied by dystrophic and destructive changes of oligodendrocytes. Damage of myelinated fibers in gray matter is present in cases with predominantly positive symptoms. Degeneration of myelinated sheaths in white matter occurs in cases of schizophrenia with predominantly negative symptoms and progress in the course of illness. Damage of myelinated fibers in white matter in schizophrenia might be followed by alterations of myelinated fibers in gray matter. Alterations of myelinated fibers and oligodendrocytes in schizophrenia might contribute abnormalities of neuronal connectivity in schizophrenia.

The present study has few limitations. First, small sample size for the study of white matter, it is necessary to repeat the study of white matter using bigger sample size. Second, we did not find the effects of neuroleptic exposure on myelin abnormalities in schizophrenia. However, imaging data suggest that neuroleptic exposure influence white matter in schizophrenia. Further studies using big sample size are needed to estimate size of myelin sheaths and frequency of small-, medium- and large-sized myelinated fibers, to study the role of astrocytes and microglia and the effects of drug therapy on white matter changes in schizophrenia, and to better understand the nature of pathological process and the presence of regeneration in white matter in schizophrenia.

The results of the present study provide evidence for morphological basis of myelin abnormalities in schizophrenia that might be useful for better understanding the pathophysiology and pathogenesis of schizophrenia and for interpretation of neuroimaging data, to develop new, more precise neuroimaging techniques of white matter visualization and to create new therapeutic strategies directed to myelin abnormalities in schizophrenia.

##  Conflict of Interests

The authors have no conflict of interest with any commercial or other associations in connection with this article.

## Figures and Tables

**Table 1:** Basic demographic characteristics (Means, SD).

Subjects	Number per group	Gender	Age (years)	PMI (hours)	Duration of illness (years)	Age at onset of illness (years)
Gray matter						

Controls	*N* = 40	29 M, 11 F	55,5 ± 14,2	5,9 ± 1,2		
Schizophrenia	*N* = 40	15 M, 25 F	58,7 ± 15,6	6,3 ± 1,8	25,1 ± 12,8	33,1 ± 14,8
SPNS	*N* = 20	6 M, 14 F	59,7 ± 16,5	5,7 ± 1,4	27,4 ± 12,5	32,2 ± 14,8
SPPS	*N* = 18	8 M, 10 F	56,6 ± 15,2	6,7 ± 2,1	21,9 ± 14,5	34,7 ± 15,4

White matter						

Controls	*N* = 12	8 M, 4 F	51,1 ± 14,0	6,5 ± 1,2		
Schizophrenia	*N* = 12	7 M, 5 F	51,1 ± 19,0	6,5 ± 1,2	27,0 ± 12,8	24,1 ± 12,6
SPNS	*N* = 6	4 M, 2 F	55,5 ± 21,0	6,1 ± 1,0	30,0 ± 14,0	25,5 ± 13,5
SPPS	*N* = 6	3 M, 3 F	46,7 ± 17,6	7,0 ± 1,3	24,0 ± 12,0	22,7 ± 12,7

SPNS: schizophrenia with predominantly negative symptoms.

SPPS: schizophrenia with predominantly positive symptoms.
